# Gait Pattern Differences between Children with Mild Scoliosis and Children with Unilateral Cerebral Palsy

**DOI:** 10.1371/journal.pone.0103095

**Published:** 2014-08-04

**Authors:** Małgorzata Domagalska-Szopa, Andrzej Szopa

**Affiliations:** 1 Institute of Medical Rehabilitation, Department of Physiotherapy, School of Health Sciences, Medical University of Silesia, Katowice, Poland; 2 Institute of Physiotherapy, Department of Physiotherapy, School of Health Sciences, Medical University of Silesia, Katowice, Poland; University of Toronto, Canada

## Abstract

This study was conducted to investigate the effects of asymmetrical body posture alone, i.e., the effects seen in children with mild scoliosis, vs. the effects of body posture control impairment, i.e., those seen in children with unilateral cerebral palsy on gait patterns. Three-dimensional instrumented gait analysis (3DGA) was conducted in 45 children with hemiplegia and 51 children with mild scoliosis. All the children were able to walk without assistance devices. A set of 35 selected spatiotemporal gait and kinematics parameters were evaluated when subjects walked on a treadmill. A cluster analysis revealed 3 different gait patterns: a scoliotic gait pattern and 2 different hemiplegic gait patterns. The results showed that the discrepancy in gait patterns was not simply a lower limb kinematic deviation in the sagittal plane, as expected. Additional altered kinematics, such as pelvic misorientation in the coronal plane in both the stance and swing phases and inadequate stance phase hip ad/abduction, which resulted from postural pattern features, were distinguished between the 3 gait patterns. Our study provides evidence for a strong correlation between postural and gait patterns in children with unilateral cerebral palsy. Information on differences in gait patterns may be used to improve the guidelines for early therapy for children with hemiplegia before abnormal gait patterns are fully established. The gait pathology characteristic of scoliotic children is a potential new direction for treating scoliosis that complements the standard posture and walking control therapy exercises with the use of biofeedback.

## Introduction

Scoliosis is primarily considered to be a structural deformation of the spine; however, the majority of patients with this diagnosis exhibit structural changes in the pelvic drop as part of the scoliotic curve [1]. Nearly all studies that have examined walking in scoliotic patients report some gait abnormality. Several studies have reported that the gait pathology depends on the severity of the spinal deformity and the type of pelvic deformity in these children [2], [3], [4]. A few studies have shown that incorrect spatial orientation of the pelvis can induce an asymmetric position and range of motion in the hip, knee, and ankle joints, thus disturbing gait patterns [5], [6].

Conversely, in children with cerebral palsy (CP), abnormal gait patterns are observed immediately, resulting from functional strategies to compensate for primary anomalies. These abnormal gait patterns are directly attributable to central nervous system damage [7], [8]. The gait patterns in children with unilateral CP share several typical features involved in the mechanism of their gait disturbances but differ inter-individually according to the extent and the location of the cerebral injury. These differences are illustrated by the variety of different classifications of hemiplegic gaits: 4 groups of hemiplegic gait patterns found by Winters et al. [9], 5 types of gait disturbances described by Hullin and coworkers [10] and 8 different gait patterns in children with hemiplegia reported by Stebbins et al. [11]. Most classifications of CP gait have been constructed using only sagittal plane data.

Children with CP often experience disruptions in postural control and subsequent postural instability [12], [13]. Most of these children present disorders of body posture. These disorders do not necessarily arise from impairments of the posture control system itself but could be the effect of other pathophysiological factors, including neurological components (paresis and paralysis) or peripheral compensatory components [14], [15].

In view of the limited number of previous studies on the effect of body posture deformities on gait patterns in children with CP, we aimed to assess and compare the effects on gait patterns of asymmetrical body posture alone, i.e., children with mild scoliosis, vs. the effects on gait patterns of body posture control impairment in children with unilateral CP.

By measuring weight bearing between the sides of the body and examining body posture (Moiré topography, MT), we previously found differences between the asymmetrical postural patterns of children with mild scoliosis and those of children with hemiplegia [16]. In addition, despite the apparent similarities among children with unilateral CP, their postural patterns differed. Depending on the weight-bearing distribution between the affected and unaffected body sides and the characteristic pelvic orientation, 2 asymmetrical postural patterns were described: 1) the pro-gravitational postural pattern (PGPP), with overloading of the affected body side, and 2) the anti-gravitational postural pattern (AGPP), with under-loading of the affected side.

Accordingly, we tested whether different postural patterns can be associated with different gait patterns in these children. We expected to find kinematic differences between the groups of children that resulted from the characteristically asymmetrical body posture. Additionally, we assumed that the discrepancy in gait pattern between children with AGPP and PGPP would include not only a lower limb kinematic deviation in the sagittal plane but also other altered kinematics that resulted from features of postural patterns, especially misorientation of the pelvis.

## Methods

The study was approved by The Ethical Committee of the Medical University of Silesia and conformed to the Helsinki Declaration. All the patients and their parents/guardians provided written informed consent prior to the study, including enrolment and data collection.

### Subjects

Forty-five children (17 girls and 28 boys) with unilateral CP participated in the study: 29 patients with right-sided deficits and 16 patients with left-sided deficits. In total, 34 patients were classified as Level I and 11 as Level II, based on the Gross Motor Function Classification System (GMFCS). The participants had a mean age of 9 years and 5 months, with a range of 7 years and 4 months to 12 years and 2 months (SD = 2.11), and they were selected from the outpatients of the local paediatric rehabilitation centres. All of these children had received a diagnosis of hemiplegia from a physician.

The group of children with mild scoliosis comprised 51 children (27 girls and 24 boys; range of lateral curvature, 11°–20°, mean, 18°; mean age, 9 years and 2 months, range, 7 years and 5 months to 12 years and 3 months (SD = 1.99). All of these children were outpatients at a local centre for corrective gymnastics.

All subjects met the following criteria: (1) older than 7 years (to minimise the incidence of unstable gait patterns), (2) ability to follow verbal directions, (3) ability to walk without assistance, and (4) no previous surgical procedures. The additional criteria for children with CP were as follows: (1) diagnosis of spastic hemiplegia, (2) no pharmacological agents at the time of the study, and (3) no spasticity management 6 months before the evaluation. The exclusion criteria were as follows: any accompanying disease that could influence the gait pattern (e.g., cardiopulmonary disorders, diabetes, or asthma) or previous hip dislocation or fracture of the lower limbs.

### Data collection and analysis

Three-dimensional instrumented gait analysis (3DGA) was performed using the Compact Measuring System for 3D Real-Time Motion Analysis (CMS-HS 3D) based on 15 active ultrasonic markers (5 triplicate ultrasound markers) with WinGait software (Zebris Medizintechnik GmbH, Germany) [17], [18].

Prior to gait analysis, the following anatomical landmarks were identified using an instrumented pointer: hip joint centre, knee rotation centre (internal and external), ankle rotation centre (internal and external), forefoot landmark (between the second and third metatarsals), and rear foot (heel). The gait data were recorded while the subjects walked on an Alfa XL treadmill (Kettler, Germany). Each child's typical over-ground walking speed (spontaneous) and time taken to walk 10 metres were collected by a single examiner before the gait analysis. Based on the spontaneous speed of walking, treadmill belt speeds were calculated as values in kilometres per hour. The children walked without shoes. Markers were attached to the skin with double-sided adhesive tape and placed bilaterally. Depending on each subject's walking ability, 5 to 8 gait cycles were recorded.

A set of 35 parameters (and their absolute values) was selected from the spatiotemporal gait parameters (speed, cadence, step length, stride length and kinematics [i.e., joint angles]) at each pelvic spatial position (pelvic tilt, pelvic obliquity, pelvic rotation), hip and knee flexion/extension, hip ab/adduction, hip rotation, and ankle plantar/dorsiflexion.

All selected gait parameters were defined according to the prior literature on gait analysis [19], [20]. These kinematics included separate values for all joint angles at one specific point in the gait cycle. Because the events during a step cycle occur regularly and can be fixed in terms of percentages, the kinematic data were collected from both lower limbs at 10%, 20% and 30% of the complete step. This interval occurs during the mid-stance phase (MST) for one leg, which constitutes approximately 10%–30% of the step cycle, during which the opposite leg is in the mid-swing phase (MSW), constituting approximately the other 70%–90% of the step cycle. Each of two experienced physical therapists selected - the most characteristic of the child - in their opinion - 3 gait cycles per subject for further analysis. The kinematic data were averaged from randomly selected 3 cycles (from all 6). The intraclass correlation coefficient (ICC), with a 95% confidence interval, was used to measure overall intraobserver and interobserver agreement. Intraobserver agreement was calculated for the pelvic obliquity angle at MST for the affected limb and the right scoliotic limb based on 2 examinations performed by the same 2 assessors in each group (children with hemiplegia and children with mild scoliosis) for 10 subjects (40 examinations in total). Interobserver agreement was calculated (for the same subjects) for the 2 assessors. For the analysis, mean ICC values of 0.80 and above reflected excellent reliability, those between 0.70 and 0.79 indicated good reliability, and those below 0.70 reflected poor to moderate reliability. The outcomes demonstrated good interobserver (0.70–0.79) and intraobserver (0.68–0.72) repeatability for both groups of subjects. Lower variability in ICC was observed for children with scoliosis than for hemiplegic children both within and between assessors.

### Statistical analysis

Non-hierarchical *k*-means clustering was used in the selection of 3DGA parameters, resulting in 3 clusters [21]. The mean and SD values for the 3DGA parameters were calculated for the entire group and for each of the 3 clusters and were compared among the subgroups. Analysis of variance (ANOVA, Tukey's post-hoc test) was used to detect differences in 3DGA among the 3 clusters. Only significant differences (*P*<0.05) among the clusters are reported. Based on the results of our previous study, which was performed on the same subjects, the children were divided into 3 subgroups according to their postural patterns [16]:

1. Children with mild scoliosis (scoliotic postural pattern, SPP)
2. Hemiplegic children with PGPP (overloaded on the affected body side)
3. Hemiplegic children with AGPP (under-loaded on the affected body side)

## Results

Using a data reduction technique, 8 grouping variables were extracted:

1. the pelvic obliquity angle at MST for the affected/normal right leg (H/R) and the unaffected/normal left leg (UH/L) at MSW,
2. the pelvic obliquity angle at MST for the UH/L leg and theH/R leg at MSW,
3. hip flexion/extension of H/R at MST,
4. the absolute value of the hip flexion/extension angle of H/R at MST,
5. the knee flexion/extension angle of H/R at MST,
6. the absolute value of the knee flexion/extension angle of H/R at MST,
7. the hip ab/adduction angle of H/R at MSW, and
8. the ankle plantar/dorsiflexion angle of H/R at MST.

In our cluster analysis results, 23 (24%) participants were classified into Cluster 1, 51 (53.1%) were included in Cluster 2, and 22 (22.9%) were included in Cluster 3 (Table 1). Three gait patterns emerged in accordance with the diagnosis, SPP, PGPP, and AGPP, and were found to clearly correspond to the cluster patterns defined as follows:

**Table 1:** Non-hierarchical *k*-means clustering.

Subgroup		Cluster 1	Cluster 2	Cluster 3	Total
SPP	(N)	0	51	0	51
	(%)	0.00%	100.00%	0.00%	53.13%
PGPP	(N)	23	0	0	23
	(%)	100.00%	0.00%	0.00%	23.96%
AGPP	(N)	0	0	22	22
	(%)	0.00%	0.00%	100.00%	22.92%
Total	(N)	23	51	22	96
	(%)	23.96%	53.13%	22.92%	100.00%

Children were diagnosed with one of the following postural patterns: SPP: scoliotic postural pattern; PGPP: pro-gravitational postural pattern; AGPP: anti-gravitational postural pattern.

1. Scoliotic gait pattern (SGP) (Cluster 2)
2. Hemiplegic pro-gravitational gait pattern (PGP) (Cluster 1)
3. Hemiplegic anti-gravitational gait pattern (AGP) (Cluster 3).

There were significant differences among the means of the various clusters for all 8 kinematics, as shown in Table 2. Table 3 shows the F values and significance levels; all differences between means are significant.

**Table 2:** Kinematics (joint angle) descriptions.

Eight grouping variables of kinematics joint angle (°)	Cluster	Mean	N	Std deviation	Minimum	Maximum
1) pelvic obliquity of H/R stance leg and UH/L swing leg	1	−2.46	23	1.86	−8.50	0.20
	2	1.32	51	2.46	−7.30	5.70
	3	7.75	22	3.28	1.40	13.20
	Total	1.89	96	4.36	−8.50	13.20
2) pelvic obliquity of UH/L stance leg and H/R swing leg	1	−3.06	23	2.68	−7.60	0.90
	2	−0.41	51	1.96	−5.80	4.60
	3	6.07	22	2.50	2.70	11.56
	Total	0.44	96	3.97	−7.60	11.56
3) stance hip flexion/extension of H/R	1	31.95	23	13.82	12.70	56.30
	2	15.39	51	4.07	10.10	24.70
	3	0.77	22	6.49	−9.80	11.90
	Total	16.00	96	13.33	−9.80	56.30
4) absolute value of stance hip flexion/extension of H/R	1	31.95	23	13.83	12.70	56.30
	2	15.30	51	4.07	10.10	24.70
	3	5.55	22	3.23	0.40	11.90
	Total	17.10	96	11.88	0.40	56.30
5) stance knee flexion/extension of H/R	1	53.37	23	7.65	35.20	70.70
	2	10.94	51	4.37	1.40	22.70
	3	0.26	22	4.06	−8.60	5.70
	Total	18.66	96	20.72	−8.60	70.70
6) absolute value of stance knee flexion/extension of H/R	1	53.37	23	7.65	35.20	70.70
	2	10.94	51	4.37	1.40	22.70
	3	3.48	22	1.97	0.20	8.60
	Total	19.40	96	20.02	0.20	70.70
7) stance ankle plantar/dorsiflexion of H/R	1	10.58	23	4.05	2.70	17.90
	2	7.40	51	2.87	0.30	14.90
	3	−5.94	22	7.29	−18.00	3.50
	Total	5.10	96	7.63	−18.00	17.90
8) stance hip ab/adduction of H/R	1	−1.96	22	5.16	−18.20	4.00
	2	0.49	23	3.87	2.60	15.50
	3	6.53	51	3.10	−1.30	13.70
	Total	5.29	96	5.63	−18.20	15.50

H: affected lower limb in children with hemiplegia or R: right lower limb in children with mild scoliosis; UH: unaffected lower limb in children with hemiplegia or L: left lower limb in children with mild scoliosis. Pelvis obliquity up (+)/down (−), hip flexion (+)/hyperextension (−), knee flexion (+)/hyperextension (−), ankle plantar (−)/dorsiflexion (+).

**Table 3:** Results of analysis of variance (ANOVA). Differences between the means of various clusters for kinematics (joint angles) are shown.

Eight grouping variables of kinematics joint angle (°)	Group	Sum of squares	*df*	Mean square	F	*P*
1) pelvic obliquity of H/R stance leg and UH/L swing leg	Between	1205.527	2	6.521	92.438	0.00000
	Within	602.764	93			
	Total	606.425	95			
2) pelvic obliquity of UH/L stance leg and H/R swing leg/L	Between	1018.445	2	5.205	97.840	0.00000
	Within	821.345	93			
	Total	484.031	95			
3) stance hip flexion/extension of H/R	Between	10973.071	2	63.614	86.247	0.00002
	Within	5486.535	93			
	Total	5916.094	95			
4) absolute value of stance hip flexion/extension of H/R	Between	8155.484	2	56.457	72.228	0.00000
	Within	4077.742	93			
	Total	5250.465	95			
5) stance knee flexion/extension of H/R	Between	38193.308	2	27.872	685.159	0.00000
	Within	19096.654	93			
	Total	2592.083	95			
6) absolute value of stance knee flexion/extension of H/R	Between	35764.104	2	27.872	714.697	0.00000
	Within	17882.052	93			
	Total	2326.905	95			
7) stance ankle plantar/dorsiflexion of H/R	Between	3643.264	2	20.354	89.496	0.00000
	Within	1821.632	93			
	Total	1892.961	95	.		
8) stance hip ab/adduction of H/R	Between	1642.690	2	14.750	55.685	0.00000
	Within	821.345	93			
	Total	1371.726	95			

H: affected lower limb in children with hemiplegia or R: right lower limb in children with mild scoliosis; UH: unaffected lower limb in children with hemiplegia or L: left lower limb in children with mild scoliosis. Pelvis obliquity up (+)/down (−), hip flexion (+)/hyperextension (−), knee flexion (+)/hyperextension (−), ankle plantar (−)/dorsiflexion (+).

Cluster 2 entirely contained the typically developing children with mild scoliosis. Cluster 1 entirely included hemiplegic children with PGPP, and cluster 3 entirely contained hemiplegic children with AGPP (Table 1).

Tukey's post-hoc test revealed that the 3DGA parameters reliably differentiated all groups: Cluster 1 was distinct from both Cluster 2 and Cluster 3, which were also distinct from each other, based on cluster means (*P*<0.001 for each comparison).

## Discussion

This study was conducted to investigate the effects of asymmetrical body posture alone, i.e., children with mild scoliosis, vs. the effects of body posture control impairment, i.e., children with unilateral CP, on gait patterns. A cluster analysis revealed 3 different gait patterns (SGP, PGP, and AGP) defined by nonoverlapping kinematics (pelvic obliquity of both stance and swing, stance hip and knee flexion/extension, stance ankle plantar/dorsiflexion and stance hip ab/adduction), which are associated with the postural patterns defined in our previous study [16].

The obtained results showed that the discrepancy in gait patterns between the groups mentioned above was not simply a lower limb kinematic deviation in the sagittal plane, as expected. Additional altered kinematics, such as pelvic misorientation in the coronal plane in both the stance and swing phases and inadequate stance phase hip ad/abduction, which resulted from features of the postural patterns, were distinguished between the 3 gait patterns.

In 3DGA, contralateral pelvic drop is identified as ipsilateral pelvic elevation [1]. For the pelvis, positive numbers indicate "up," and negative numbers indicate "down" (−4° to 4° in normal gait) [1]. Abnormal pelvic motion in the coronal plane is displayed as excessive action (pelvic hike or pelvic drop), and inadequate pelvic motion is observed as fixation [1].

In the SGP, inadequate pelvic motion was observed during both MST and MSW. Static misalignment of the pelvis, such as contralateral or ipsilateral drop (negative or positive number, depending on the type of spinal deformation), may result in the motion described above and in deviations from the normal gait. These observations were confirmed by observing the stance hip in the coronal plane and its associated kinematics. This approach is appropriate because hip ad/abduction is measured as the motion of the femur relative to the pelvis. Pelvic obliquity secondary to scoliosis leads to excessive abduction in the hip on the low side, and the hip on the high side will show excessive adduction [1]. Thus, in the gait of children with scoliosis, excessive abduction in the hip on the low side of the pelvis and, conversely, excessive adduction on the high side of the pelvis can have average values of almost zero with regard to stance hip kinematics.

A similar tendency was observed in AGP, but the pelvic obliquity of the affected stance limb (hemi-pelvis up) was, on average, 3 times higher than that in SGP and showed a change greater than 6 times the swing of the affected limb. The opposite misorientation of the pelvis was characteristic for PGP: ipsilateral pelvic drop and contralateral pelvic hike such that the stance hemi-pelvis was low and the swing non-hemi-pelvis was high. Additionally, in most cases, contralateral trunk lean was assisted by pelvic hiking (Duchenne's sign). Furthermore, in both hemiplegic gait patterns, the pelvis did not change its orientation in the coronal plane during swing. The hemiplegic side became high in AGP, and the hemiplegic side became low in PGP. This alteration most likely results from ipsilateral adductor contracture or spasticity, which are typical in children with AGP, and from gait compensation for a short hip abduction lever in children with PGP. The excessive adduction within the hip joint in AGP offers further proof of ipsilateral adductor contracture or spasticity, whereas the opposite hip abduction patterns can confirm lever-arm dysfunction (LAD) in children with PGP [1], [7].

As expected, clear differences in the sagittal plane kinematics of the hip, knee, and ankle at MST were observed between the scoliotic and hemiplegic gait patterns. Whereas the kinematics of the hip, knee, and ankle at stance in children with scoliosis oscillated within the normal range of values, 2 pathological but opposing sets of sagittal plane kinematic values for the hip, knee, and ankle were noted in children with hemiplegia. The 2 gait patterns, AGP and PGP, contrast with each other due to the sagittal plane kinematics of the lower limb joints. These differences in gait deviation can be summarised as 1) an excessive extension (insufficient hip flexion, insufficient knee flexion, or hyperextension with ankle plantar flexion) of the stance limb for AGP and 2) mass flexion (inadequate hip extension and excessive knee flexion with ankle dorsi-flexion) of the stance limb typical in PGP.

This study shows that postural patterns affect the gait pattern in both children with mild scoliosis and children with unilateral CP. Additionally, our results show that a few deviations in the scoliotic gait, including inadequate pelvic motion in the coronal plane at stance and swing and inadequate stance hip ad/abduction, depended upon the postural pattern of children with scoliosis.

Although the majority of common gait problems in hemiplegic children involve a kinematic deviation in the sagittal plane [9]–[11], [22], [23], our study has identified several other unreported kinematic deviations in the hemiplegic gait that result from features of hemiplegic postural patterns. We reported not only deviations related to the release of the hemi-limb during the swing phase but also a lack of stability during the stance phase (i.e., impairment of central body control and postural pattern). We recognised and defined 2 different gait patterns in children with unilateral CP. The current study, to our knowledge, is the first to define the hemiplegic gait patterns (AGP and PGP) in relation to their postural patterns.

However, these patterns might be the only compensatory strategy possible or may represent a pathological adaptation that has to be corrected. A few symptoms that are potentially important for the treatment decision-making process and that pose a risk that pathological adaptation will develop were observed during the present study. The most typical patterns for AGP were under-loading of the affected side during walking and hemi-pelvis up with fixed equinovarus during both the support and the swing phases. It appears that the essential part of this pathological mechanism is excessive pushing from the forefoot of the affected foot to the ground, caused perhaps by an inability to process and perceive stimuli on one side of the body due to a lack of proprioception, similar to hemispatial neglect [24]. Permanent plantar flexion of the affected foot is dangerous because it produces m. soleus contraction and subsequent shortening of the Achilles tendon and foot deformities. Treatment for children with AGP should consist of finding ways to direct the patient's attention to the affected leg (including sensory integration and techniques with weight bearing), usually conducted incrementally from a kneeling to a standing position and ultimately during walking. Additionally, spasticity management and contracture management, such as inhibiting casts and botulinum toxin (TBA) injections, should be used [25], [26].

The pathological adaptation mechanism characteristic of PGP was accompanied by many more dangerous features. These features result primarily from overloading of the affected body side and consist of an unstable hip, knee and foot. This condition is primarily caused by the hemi-pelvis dropping in the stance phase and generates a dangerous stereotypy, e.g., adduction and internal rotation of the hip, with the danger of hip subluxation or luxation. Treatment of children with PGP requires orthotic management, such as AFO (Ankle Foot Orthosis) or Dynamic Ankle Foot Orthosis (DAFO) [27].

Management of the treatment of children with hemiplegia without considering the differences between AGP and PGP may produce an irreparable loss of the ability to maintain a standing position and to walk independently. Early treatment of children with hemiplegia requires, from the beginning, a proper approach to the predominant primary postural and motor problems as well as the anticipation of secondary problems such as mechanisms of pathological adaptation. We hope that recognising 2 different postural and gait patterns in children with hemiplegia will be an essential step towards developing a management algorithm for physiotherapy in our follow-up studies.

### Study limitations

The number of gait parameters selected for this study was limited for spatiotemporal parameters and kinematics to 10%, 20%, and 30% of the step cycle, which approximately represents the MST (single support phase) for the lower limb and, simultaneously, the MSW for the opposite leg, which constitutes 90%, 80%, and 70%, respectively, of the step cycle. Based on our previous study,^6^ we selected these gait parameters, which can be related to postural parameters according to the differing asymmetry of the body posture in children with scoliosis and children with unilateral CP, especially as a result of the asymmetrical position of the pelvis during standing. Therefore, the selected parameters described the gait pattern of both lower limbs in the same phase of the step cycle (MST and MSW) and can serve as a good representation of both pathological scoliotic and hemiplegic gaits. Such an approach makes it difficult to compare the obtained results with those found in the previous literature on gait analysis.

### Conclusions

In conclusion, our findings indicate that the discrepancy in scoliotic and hemiplegic gait patterns results not only from a lower limb kinematic deviation in the sagittal plane but also from altered kinematics that result from features of the postural patterns. The results of the gait analysis may be applied to modify rehabilitation programs and adjust them to meet the individual needs of patients with scoliosis and hemiplegia. The gait pathology characteristic of scoliotic children is a potential new direction for treating scoliosis that complements the standard posture and walking control therapy exercises with the use of biofeedback.

Our study provides evidence of a strong correlation between postural and gait patterns in children with unilateral CP. Information on differences in the posture and gait may improve the guidelines for early therapy for children with hemiplegia before the abnormal gait patterns are fully established.
